# Supplementary material for: ProteinShader: illustrative rendering of macromolecules
Source: BMC Struct Biol. 2009 Mar 30;9:19. doi: 10.1186/1472-6807-9-19 (PMC2672931; doi:10.1186/1472-6807-9-19)
Supplement: Additional file 1 — ProteinShader program without source code. This compressed file contains the complete ProteinShader program including associated libraries, but no source code. A README.txt file gives an overview of the ProteinShader distribution, and the index.html file in the help subdirectory has directions on getting started with the program as well as a set of tutorials. [file 1472-6807-9-19-S1.zip › ProteinShader-beta-0_9_4-binary/help/api/org/proteinshader/structure/enums/SideChainEnum.html]

SideChainEnum (ProteinShader API)


|  |  |  |
| --- | --- | --- |
| **Overview** \| **Package** \| **Class** \| **Use** \| **Tree** \| **Deprecated** \| **Index** \| **Help** | |  |
| **PREV CLASS**   **NEXT CLASS** | **FRAMES**    **NO FRAMES**     **All Classes** |  |
| SUMMARY: NESTED \| ENUM CONSTANTS \| FIELD \| METHOD | DETAIL: ENUM CONSTANTS \| FIELD \| METHOD |  |


---


## org.proteinshader.structure.enums Enum SideChainEnum

```
java.lang.Object
  java.lang.Enum<SideChainEnum>
      org.proteinshader.structure.enums.SideChainEnum
```

**All Implemented Interfaces:**: Serializable, Comparable<SideChainEnum>

---

``` public enum SideChainEnum extends Enum<SideChainEnum> ```

Provides an enumeration to specify how an amino
acid side chain should be displayed is it is shown
in combination with a tube or ribbon segment.

---

**Enum Constant Summary**

`BALLS_AND_STICKS`
  
          Use a balls-and-sticks representation.

`NONE`
  
          Show nothing.

`SPACE_FILLING`
  
          Use a space filling representation (spheres only).

`STICKS`
  
          Use sticks (thin cylinders) only.


| **Method Summary** | |
| --- | --- |
| `static SideChainEnum` | `getDefaultSideChainType()`             Returns the default side chain type, which is NONE. |
| `String` | `getMenuName()`             Returns the name of the SideChainEnum in a form suitable for use in a menu. |
| `String` | `toString()`             Returns the name of the SideChainEnum in a form suitable for use in a menu. |
| `static SideChainEnum` | `valueOf(String name)`             Returns the enum constant of this type with the specified name. |
| `static SideChainEnum` | `valueOfMenuName(String menuName)`             Returns the SideChainEnum with the same menu name as the String given as an argument. |
| `static SideChainEnum[]` | `values()`             Returns an array containing the constants of this enum type, in the order they're declared. |

**Methods inherited from class java.lang.Enum**

`clone, compareTo, equals, getDeclaringClass, hashCode, name, ordinal, valueOf`

**Methods inherited from class java.lang.Object**

`finalize, getClass, notify, notifyAll, wait, wait, wait`

**Enum Constant Detail**

### NONE

```
public static final SideChainEnum NONE
```

:   Show nothing.
    The menu name for this enum is "None".

---


### SPACE\_FILLING

```
public static final SideChainEnum SPACE_FILLING
```

:   Use a space filling representation (spheres only).
    The menu name for this enum is "Space Filling".

---


### BALLS\_AND\_STICKS

```
public static final SideChainEnum BALLS_AND_STICKS
```

:   Use a balls-and-sticks representation.
    The menu name for this enum is "Balls and Sticks".

---


### STICKS

```
public static final SideChainEnum STICKS
```

:   Use sticks (thin cylinders) only.
    The menu name for this enum is "Sticks".


**Method Detail**

### values

```
public static final SideChainEnum[] values()
```

:   Returns an array containing the constants of this enum type, in
    the order they're declared. This method may be used to iterate
    over the constants as follows:

    ```
    for(SideChainEnum c : SideChainEnum.values())
            System.out.println(c);
    ```

    :   **Returns:**: an array containing the constants of this enum type, in the order they're declared

---


### valueOf

```
public static SideChainEnum valueOf(String name)
```

:   Returns the enum constant of this type with the specified name.
    The string must match *exactly* an identifier used to declare an
    enum constant in this type. (Extraneous whitespace characters are
    not permitted.)

    :   **Parameters:**: `name` - the name of the enum constant to be returned. **Returns:**: the enum constant with the specified name **Throws:**: `IllegalArgumentException` - if this enum type has no constant with the specified name

---


### getDefaultSideChainType

```
public static SideChainEnum getDefaultSideChainType()
```

:   Returns the default side chain type, which is NONE.

    :   **Returns:**: The default side chain type.

---


### getMenuName

```
public String getMenuName()
```

:   Returns the name of the SideChainEnum in a form suitable for use
    in a menu.

    :   **Returns:**: The SideChainEnum as a String.

---


### toString

```
public String toString()
```

:   Returns the name of the SideChainEnum in a form suitable for use
    in a menu.

    :   **Overrides:**: `toString` in class `Enum<SideChainEnum>`
    :   **Returns:**: The SideChainEnum as a String.

---


### valueOfMenuName

```
public static SideChainEnum valueOfMenuName(String menuName)
```

:   Returns the SideChainEnum with the same menu name as the String
    given as an argument.

    :   **Returns:**: The SideChainEnum matching the menu name. **Throws:**: `IllegalArgumentException` - if the menu name does not match a SideChainEnum.


---


|  |  |  |
| --- | --- | --- |
| **Overview** \| **Package** \| **Class** \| **Use** \| **Tree** \| **Deprecated** \| **Index** \| **Help** | |  |
| **PREV CLASS**   **NEXT CLASS** | **FRAMES**    **NO FRAMES**     **All Classes** |  |
| SUMMARY: NESTED \| ENUM CONSTANTS \| FIELD \| METHOD | DETAIL: ENUM CONSTANTS \| FIELD \| METHOD |  |


---

# *Copyright © 2007-2008*
